# A MYLK variant regulates asthmatic inflammation via alterations in mRNA secondary structure

**DOI:** 10.1038/ejhg.2014.201

**Published:** 2014-10-01

**Authors:** Ting Wang, Tong Zhou, Laleh Saadat, Joe GN Garcia

**Affiliations:** 1 Arizona Respiratory Center and Department of Medicine, University of Arizona, Tucson, AZ, USA

## Abstract

Myosin light-chain kinase (*MYLK*) is a gene known to be significantly associated with severe asthma in African Americans. Here we further examine the molecular function of a single-nucleotide polymorphism (SNP), located in the non-muscle myosin light-chain kinase isoform (nmMLCK), in asthma susceptibility and pathobiology. We identified nmMLCK variant (reference SNP: rs9840993, NM_053025: 721C>T, c.439C>T) with a distinct mRNA secondary structure from the other variants. The nmMLCK variant (721C) secondary structure exhibits increased stability with an elongated half-life in the human endothelial cell, and greater efficiency in protein translation initiation owing to an increased accessibility to translation start site. Finally, nmMLCK expression of 721C- and 721T-containing *MYLK* transgenes were compared in nmMLCK^−/−^ mice and confirmed deleterious effects of nmMLCK expression on asthmatic indices and implicated the augmented influence of *MYLK* 721C>T (c.439C>T) SNP on asthma severity. The confirmation of the novel mechanism of the regulation of asthmatic inflammation by a *MYLK* advances knowledge of the genetic basis for asthma disparities, and further suggests the potential of nmMLCK as a therapeutic target. Our study suggests that in addition to altering protein structure and function, non-synonymous SNPs may also lead to phenotypic disparity by altering protein expression.

## Introduction

Within a rising US and world-wide prevalence of asthma, there is strong evidence for ethnic disparities in asthma susceptibility and severity with greater mortality,^1^ more severe obstruction and greater number of severe attacks occurring in asthmatics of African descent (AD).^2^ We recently explored *MYLK*, a gene encoding the Ca^2+^/calmodulin-dependent myosin light-chain kinase (MLCK), as a candidate gene for asthma susceptibility^3^ taking advantage of extensive gene re-sequencing in samples from Europeans and African Americans.^4^ We identified the significant association of an African-specific non-synonymous variant in *MYLK* (reference single-nucleotide polymorphism (SNP): rs9840993, NM_053025: 721C>T, p.Pro147Ser or c.439C>T) with severe asthma.^3^ The human *MYLK* gene encodes three isoforms including non-muscle MLCK isoform (nmMLCK), smooth muscle isoform (smMLCK) and telokin (KRP), a small myosin filament-binding protein. Both smMLCK and nmMLCK phosphorylate myosin light chains to regulate cellular contraction and relaxation.^5^ smMLCK has been well studied in the pathogenesis of asthma as a key contributor to airway smooth muscle contractile function remodeling, characteristic of the asthmatic phenotype.^6^ In contrast, limited information is known about the role of the nmMLCK isoform in asthma pathobiology. The identified asthma susceptibility *MYLK* variant (NM_053025: 721C>T, p.Pro147Ser or c.439C>T) within the unique N terminus of nmMLCK, residing at significant distance from the smMLCK start site at 922aa.^3^ Consistent with a potential role for nmMLCK in asthma pathobiology, our structure/function studies in non-muscle tissues, such as gastrointestinal epithelium and lung vascular endothelium, have underscored a key role for nmMLCK in inflammatory responses wherein nmMLCK regulates vascular integrity (via interplay of cell contractile forces and cell–cell/cell–matrix contacts) and leukocyte influx into lung tissues.^7,8^ We recently reported that protein levels of nmMLCK correlate with experimental asthma susceptibility and severity.^9^ In this report, we analyzed the functionality of the SNP 721C>T (c.439C>T), which is strongly associated with severe asthma in African Americans, and identified a novel aspect of mRNA secondary structure.

## Materials and Methods

We calculated RNA folding energies (Δ*G*) using the RNAfold^10,11^ and mfold program.^12^ The recombinant nmMLCK1 constructs (721T or 721C) were transfected into human endothelial cells for mRNA decay and protein expression analysis. In addition, these plasmids were used to transfect the mouse lung in a model of OVA-induced experimental asthma. Asthmatic inflammatory parameters were analyzed as described previously.^9^ Detailed methods are in online methods session.

## Results and discussion

**Figure 1:**
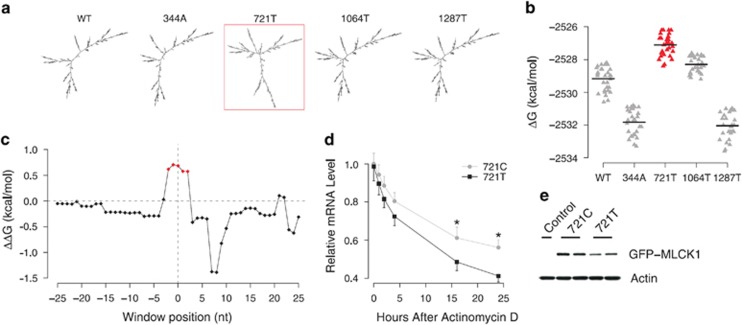
mRNA secondary structure affects *MYLK* gene translation efficiency. (**a**) mRNA secondary structures of MFE for wild-type (WT) *MLCK* gene and its variants (344A, 721T, 1064T and 1287T). (**b**) Free energy of the top 30 optimal/suboptimal mRNA secondary structures for each MYLK variants. The solid line depicts the mean of 30 values. (**c**) Landscape of the local accessibility gap (ΔΔ*G*) between the secondary structures of 721T and 721C around the start codon. ‘0' on *x* axis indicates the position of the start codon. The local accessibility was calculated using a sliding window of three nucleotides in length and one nucleotide in step. The windows with ΔΔ*G* >0.5 kcal/mol were highlighted in red. (**d**) Relative mRNA decay curve of MLCK1-GFP (721T or 721C) in HLMVECs after actinomycin D exposure (5 μg/ml). *P<0.05. (**e**) Expression levels of MLCK1-GFP (721T or 721C) protein in human lung ECs 48 h after transfection.

Within the four identified coding SNPs in nmMLCK in our previous study (Supplementary Table 1),^3^ rs9840993 (NM_053025: 721C>T, c.439C>T) is the only SNP significantly associated with severe asthma (the corresponding variant information has been deposited in the LOVD 3.0 database: http://databases.lovd.nl/shared/variants/0000040191). This is also an AD-specific SNP^3^ with very low minor allelic frequencies in European decent individuals. To analyze the global and local mRNA stability of each variant, we calculated RNA folding energies (Δ*G*) using both the *RNAfold* program in the Vienna package^10,11^ and *mfold* program^12^ based on NCBI reference sequence NM_053025 (ancestral allele of each SNP was used to define the wild-type (WT) mRNA sequence). *In silico* prediction by *RNAfold* demonstrated that compared with other *MYLK* variants, the 721T variant greatly changes the global mRNA secondary structure of minimum free energy (MFE; Figure 1a and Supplementary Figure S1). The MFE of 721T is higher than all other variants (WT, 344A, 1064T and 1287T; Supplementary Figure S2). As expected, a similar trend was obtained when the *mfold* program is applied. We used *mfold* to predict the top 30 optimal and suboptimal foldings of each variant. The free energy of the optimal and suboptimal foldings of 721T is significantly higher than that of all the other variants (*t*-test: *P*<10^−10^) (Figure 1b and Supplementary Figure S3). In comparison with MFE structure, RNA centroid structure of the ensemble was reported to produce 30% fewer prediction errors. Here we also computed the centroid structures of WT and 721T by *RNAfold*^13^ and found that the folding energy of the centroid structure of 721T is higher than that of WT. The free energy gap (ΔΔ*G*) between 721T and WT is >4 kcal/mol. These results suggest that the global structure of 721T variant is the most unstable compared with other *MYLK* variants and thus is more likely to be degraded.^14, 15, 16^

Increasing evidences suggest that reduced mRNA local stability near the translation initiation region may lead to increased translation efficiency.^17, 18, 19^ Therefore, we further analyzed the mRNA local accessibility (free energy required to open local structure) around the translation start codon using a sliding window of three nucleotides in length and one nucleotide in step. Interestingly, we found that there are five clustered windows with lower accessibility in 721T comparing with 721C (Figure 1c). The ΔΔ*G* between 721T and 721C is >0.5 kcal/mol for each of the five continuous windows, which can be rarely observed along the nmMLCK mRNA (Supplementary Figure S4). We also checked the probability of observing five windows with the sum of ΔΔ*G* larger than that of the five clustered windows around start codon by randomly selecting the nucleotides within nmMLCK mRNA. Among the 10 000 time randomization, we failed to observe such a pattern (Supplementary Figure S5), indicating that the clustered windows with decreased accessibility around start codon in 721T do not reflect random chance but rather suggest that the translation efficiency of 721C is potentially higher than that of 721T.

To further validate our findings, we assayed and compared mRNA stability and translational efficiency of variants of 721C and 721T. EGFP-labeled nmMLCK constructs with 721C or 721T were ectopically transfected and expressed in human endothelial cells to compare the rate of mRNA decay. Twenty four hours after the transfection, transcription was inhibited with actinomycin D (5 *μ*g/ml), and the levels of nmMLCK-GFP transcripts at 0–24 h were determined by real-time PCR. Actinomycin D significantly reduced mRNA levels by both constructs, while the relative mRNA level by 721C variant is significantly higher than that of 721T (Figure 1d). These observations indicate that 721C variant mediates an increased mRNA stability and reduced intracellular decay. Protein levels of nmMLCK-GFP were determined in these ECs without actinomycin D treatment (Figure 1e). Our results demonstrated that mutating nmMLCK at position 721 from T to C recapitulates higher expression levels of protein, indicating that protein translation is more efficient with the variant 721C.

**Figure 2:**
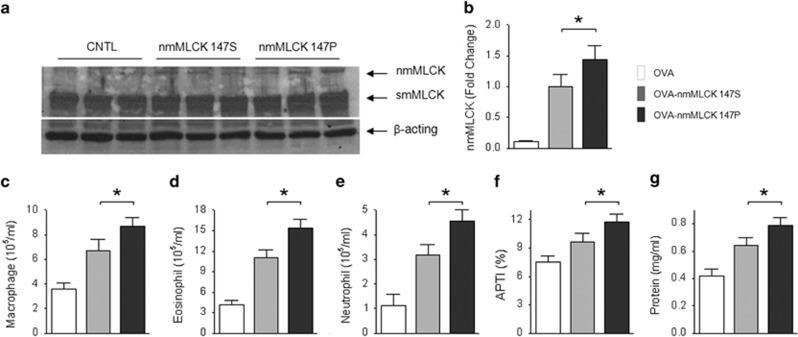
Effect of overexpressed human nmMLCK1 variants (147P or 147S) on asthmatic inflammation in a murine model. OVA-challenged nmMLCK^−/−^ mice received nmMLCK1 express transgene to overexpress nmMLCK1 (147P or 147S). (**a**) Representative nmMLCK1 overexpression in mouse lung tissues after transfection. (**b**) Quantified relative levels of expressed nmMLCK variants. (**c**–**e**) Effect of nmMLCK1 variant overexpression on inflammatory leukocyte infiltration (macrophage, eosinophil and neutrophil). (**f**) Effects of nmMLCK1 variant overexpression on airway hyper-reactivity reflected by acetylcholine-induced APTI. (**g**) Effect of nmMLCK1 variant overexpression on BAL protein levels. *P<0.05 between groups of nmMLCK1 147P and 147S. *n*=5–6.

To verify the functional effects of 721C variant (147Pro) in contributing to asthmatic susceptibility and severity, we reversely expressed human nmMLCK1 in the pulmonary endothelium of nmMLCK^−/−^ mice utilizing the ACE antibody-tagged liposome delivery system used to deliver MLCK expressing plasmids. This gene delivery system is consistently targeting pulmonary endothelium with to overexpress nmMLCK1 in mouse lung endothelium (Supplementary Figure S6). nmMLCK1 overexpression in mouse lung tissues with the nmMLCK variant 721C (147Pro) exhibited higher expression efficiency than that of the 721T variant (147Ser or WT; Figure 2a and b), consistent with the findings in computation analysis and endothelial cell *in vitro* assays. As expected, nmMLCK1 overexpression augmented asthmatic inflammatory parameters including airway hyper-responsiveness (Figure 2c), with the disease-associated 721C variant eliciting stronger allergic inflammation than the *MYLK* variant 721T (Figure 2c). Similar effects were observed in other inflammatory parameters such as airway inflammatory leukocyte infiltration (Figure 2d–f) and BAL protein leakage (Figure 2g). These data are consistent with findings of upregulation of nmMLCK transcripts in both human or murine asthmatic subjects (Supplementary Figure S7). These studies represent the first characterization of a biological function of a *MYLK* SNP *in vivo*.

In summary, our studies underscore the contributory role of *MYLK* genetic variants to asthma susceptibility in populations of African ancestry, and highlight the role of nmMLCK expression, especially in lung endothelium, to asthma pathogenesis. Although the link between *MYLK* variants, expression of specific MLCK isoforms and asthma appears to be certain, additional studies are required to establish a mechanistic relationship between genetic variants and functional alterations of this interesting gene product. Moreover, although *MYLK* variants may account for severe symptoms in African American asthmatics, further association studies, analyzing additional nearby SNPs and independent samples are required.
