# Supplementary material for: A MYLK variant regulates asthmatic inflammation via alterations in mRNA secondary structure
Source: Eur J Hum Genet. 2014 Oct 1;23(6):874–6. doi: 10.1038/ejhg.2014.201 (PMC4795064; doi:10.1038/ejhg.2014.201)
Supplement: Supplementary Methods [file ejhg2014201x1.doc]

***Online Methods***

**A MYLK Variant Regulates Asthmatic Inflammation via Alterations in mRNA Secondary Structure**

Ting Wang^*^, Tong Zhou^*^, Laleh Saadat, Joe G. N. Garcia

Arizona Respiratory Center and Department of Medicine, University of Arizona, Tucson, AZ

*,equally contributed first authors.

**Running title:** A MYLK Variant Regulates Asthmatic Inflammation

**Funding Source:** Supported by National Institutes of Health Grants HL91899 and HL58064 (JGNG).

**Conflict of interest:** The authors have no conflict of interest to declare.

**Key Words:** lung inflammation, nmMLCK, SNP

**Address correspondence to:**

Joe G. N. Garcia, MD

Senior Vice President for Health Sciences

University of Arizona

Drachman Hall, Room B-207

1295 North Martin Avenue

P.O. Box 210202

Tucson, AZ 85721-0202

(520) 626-1197

[skipgarcia@email.arizona.edu](mailto:skipgarcia@email.arizona.edu)

**Online Methods**

**mRNA secondary structure prediction** We calculated RNA folding energies (ΔG) using the *RNAfold* program in the Vienna package ^1,2^ and *mfold* program ^3^.

**Cell culture.** Human pulmonary artery endothelial cells (EC) obtained from Lonza (Basel, Switzerland) were cultured in endothelial growth medium-2 (EGM-2) as we have previously described ^4^. Endothelial cells were utilized at passages 6–7 and on the day prior to experimentation. The nmMLCK1 constructs (721T or 721C) with GFP tag ^5^ were transfected with X-fect system (clontech, Mountain View, CA) following manufacture’s menu. The cells were examined under fluorescent microscope 24 hr post transfection to determine the transfection efficiency, and used for mRNA stability analysis ^6^ or western blot to determine the protein levels.

**Animal care and procedures.** Genetically engineered murine line nmMLCK^-/-^ mice and wild type C57BL6 mice (Jackson Laboratories, Bar Harbor, ME) were used for this study. All animal procedures were approved by Institutional Animal Care and Use Committee. Experimental murine asthma was induced by ovalbumin (OVA) administration as previously described ^7^.

**Bronchoalveolar lavage (BAL) fluid.** The pulmonary vasculature was perfused clear via the pulmonary artery with sterile phosphate-buffered saline (PBS). BAL was performed and analyzed as previously described ^7^.

**Statistical analysis.** For all *in vivo* studies reported, values are reported as mean ± SEM. Two-way ANOVA is used to determine *P*-value and significance of alteration. A *P*-value of less than 0.05 was considered significant. The *in vivo* statistical analysis was done using OriginPro 7.5 (OriginLab Corporation, Northampton, MA) software.

1. Hofacker IL, Fontana W, Stadler PF, Bonhoeffer LS, Tacker M, Schuster P: Fast folding and comparison of RNA secondary structures. *Monatshefte für Chemie* 1994; **125:** 167-188.

2. Hofacker IL, Stadler PF: Memory efficient folding algorithms for circular RNA secondary structures. *Bioinformatics* 2006; **22:** 1172-1176.

3. Zuker M, David H. Mathews DH, Turner DH: Algorithms and Thermodynamics for RNA Secondary Structure Prediction: A Practical Guide; in: Barciszewski J, Clark BFC (eds): *RNA Biochemistry and Biotechnology*. Dordrecht, NL: Kluwer Academic Publishers, 1999, pp 11-43.

4. Wang T, Chiang ET, Moreno-Vinasco L *et al*: Particulate matter disrupts human lung endothelial barrier integrity via ROS- and p38 MAPK-dependent pathways. *Am J Respir Cell Mol Biol* 2010; **42:** 442-449.

5. Brown M, Adyshev D, Bindokas V, Moitra J, Garcia JG, Dudek SM: Quantitative distribution and colocalization of non-muscle myosin light chain kinase isoforms and cortactin in human lung endothelium. *Microvasc Res* 2010; **80:** 75-88.

6. Wang G, Guo X, Floros J: Differences in the translation efficiency and mRNA stability mediated by 5'-UTR splice variants of human SP-A1 and SP-A2 genes. *Am J Physiol Lung Cell Mol Physiol* 2005; **289:** L497-508.

7. Wang T, Moreno-Vinasco L, Huang Y *et al*: Murine lung responses to ambient particulate matter: genomic analysis and influence on airway hyperresponsiveness. *Environ Health Perspect* 2008; **116:** 1500-1508.
