# Supplementary material for: A MYLK variant regulates asthmatic inflammation via alterations in mRNA secondary structure
Source: Eur J Hum Genet. 2014 Oct 1;23(6):874–6. doi: 10.1038/ejhg.2014.201 (PMC4795064; doi:10.1038/ejhg.2014.201)
Supplement: Supplementary Information [file ejhg2014201x2.pdf]

Supplement Table 1

	Function	mRNA Position	Allele	Protein Position	Residue
**rs28497577**	missense	344	CCC⇒CAC	21	P [Pro]⇒H [His]
**rs4678047**	synonymous	1287	ACC⇒ACT	335	T [Thr]⇒T [Thr]
**rs3796164**	missense	1064	GCG⇒GTG	261	A [Ala]⇒V [Val]
**rs9840993**	missense	721	CCA⇒TCA	147	P [Pro]⇒S [Ser]

Supplement Figure S1

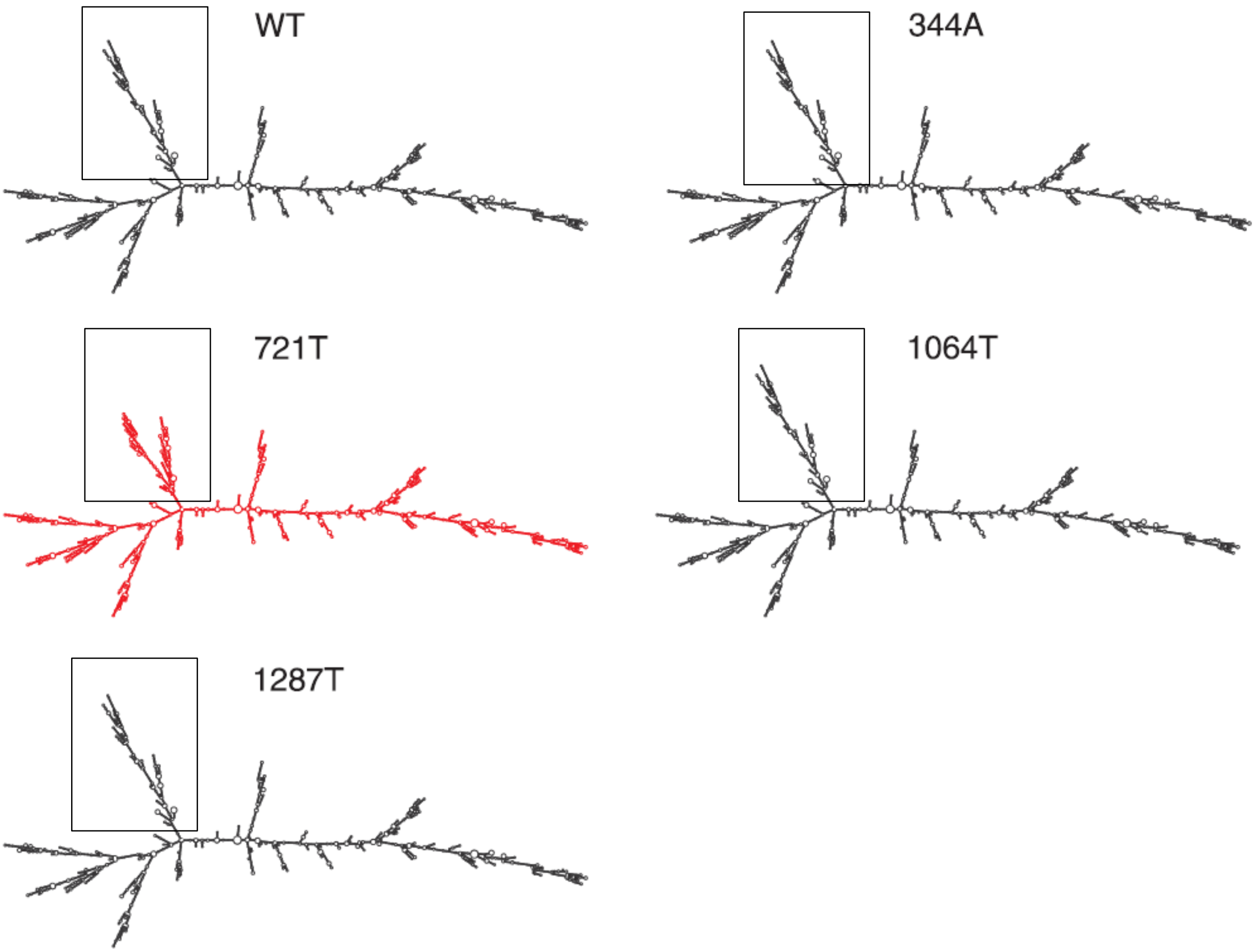

Figure S1. mRNA MFE secondary structure for wildtype (WT) MLCK gene and its mutants (344A, 721T, 1064T, and 1287T). Here, the mRNA only includes coding region.

Supplement Figure S2

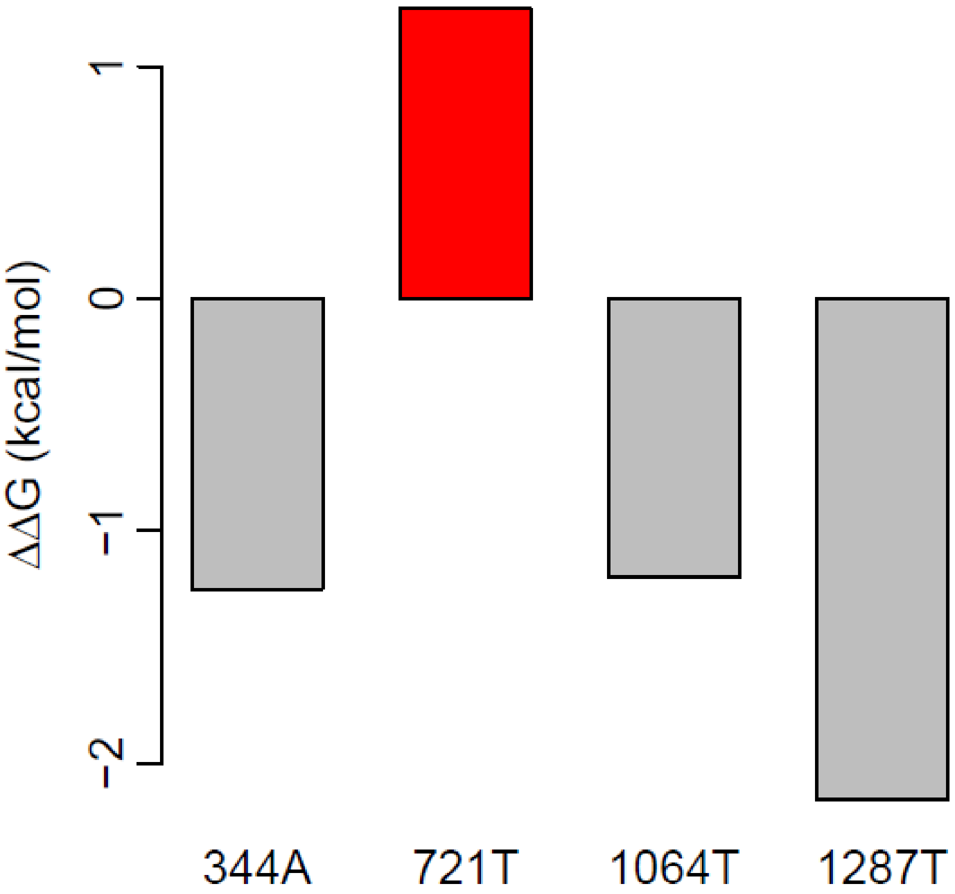

Figure S2. Free energy gap (ΔΔG) of mRNA MFE secondary structure between the mutated and wildtype nmMLCK mRNAs. The mutants include 344A, 721T, 1064T, and 1287T. MFE was calculated by the RNAfold program in the Vienna Package.

Supplement Figure S3

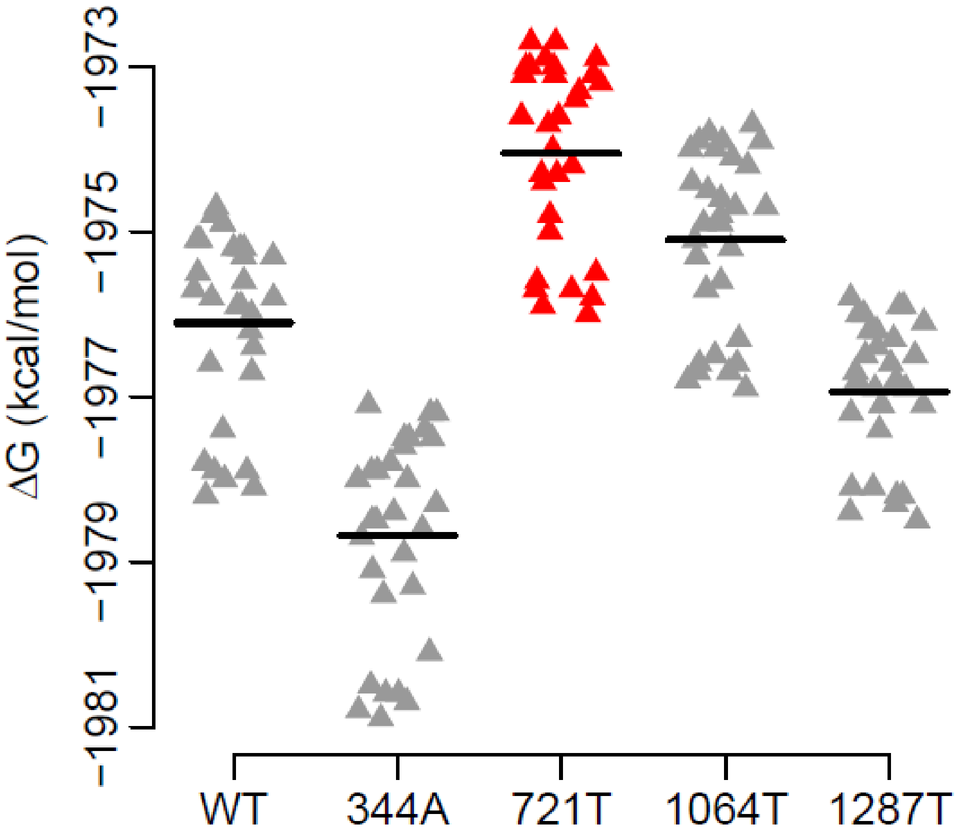

Figure S3. Free energy of the top 30 optimal/suboptimal mRNA secondary structures for each nmMYLK variants. The solid line depicts the mean of thirty values. Here, the mRNA only includes coding region.

Supplement Figure S4

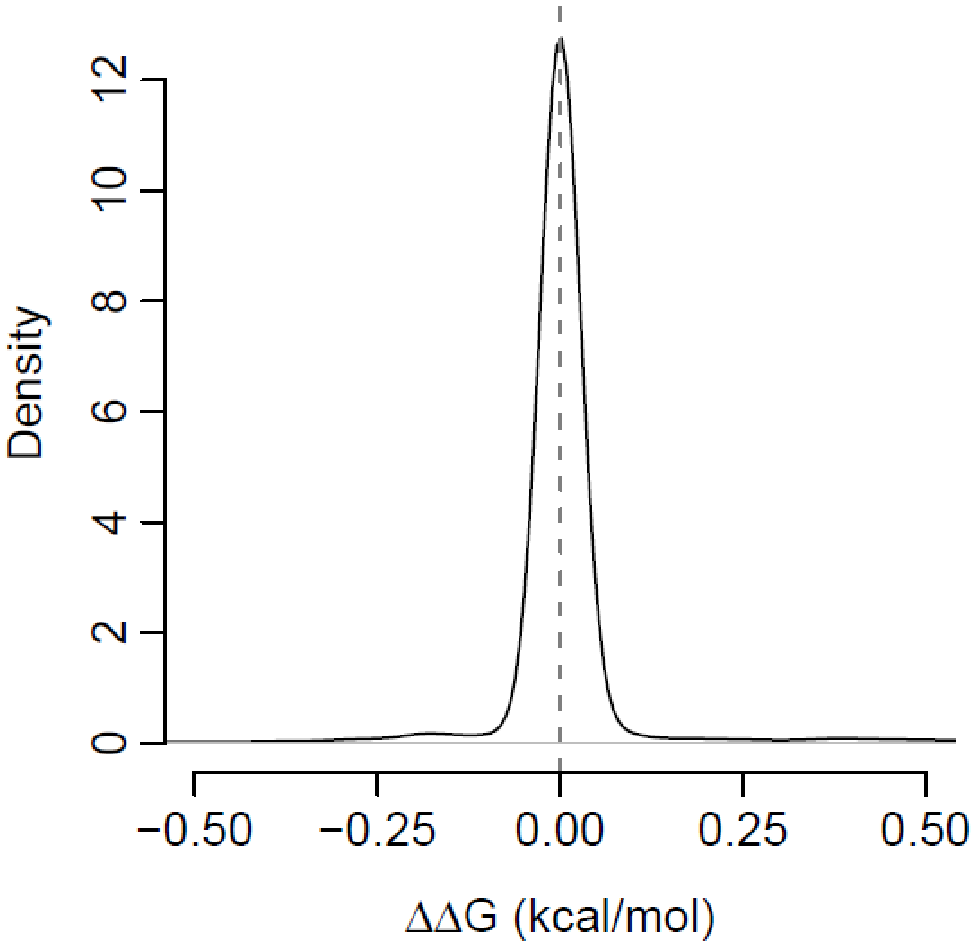

Figure S4. The distribution of local accessibility gap (ΔΔG) between the mRNA secondary structures of 721T and 721C. The local accessibility was calculated using a sliding window of 3 nucleotides in length and 1 nucleotide in step along the mRNA sequence. The windows with ΔΔG > 0.5 kcal/mol can be rarely observed.

Supplement Figure S5

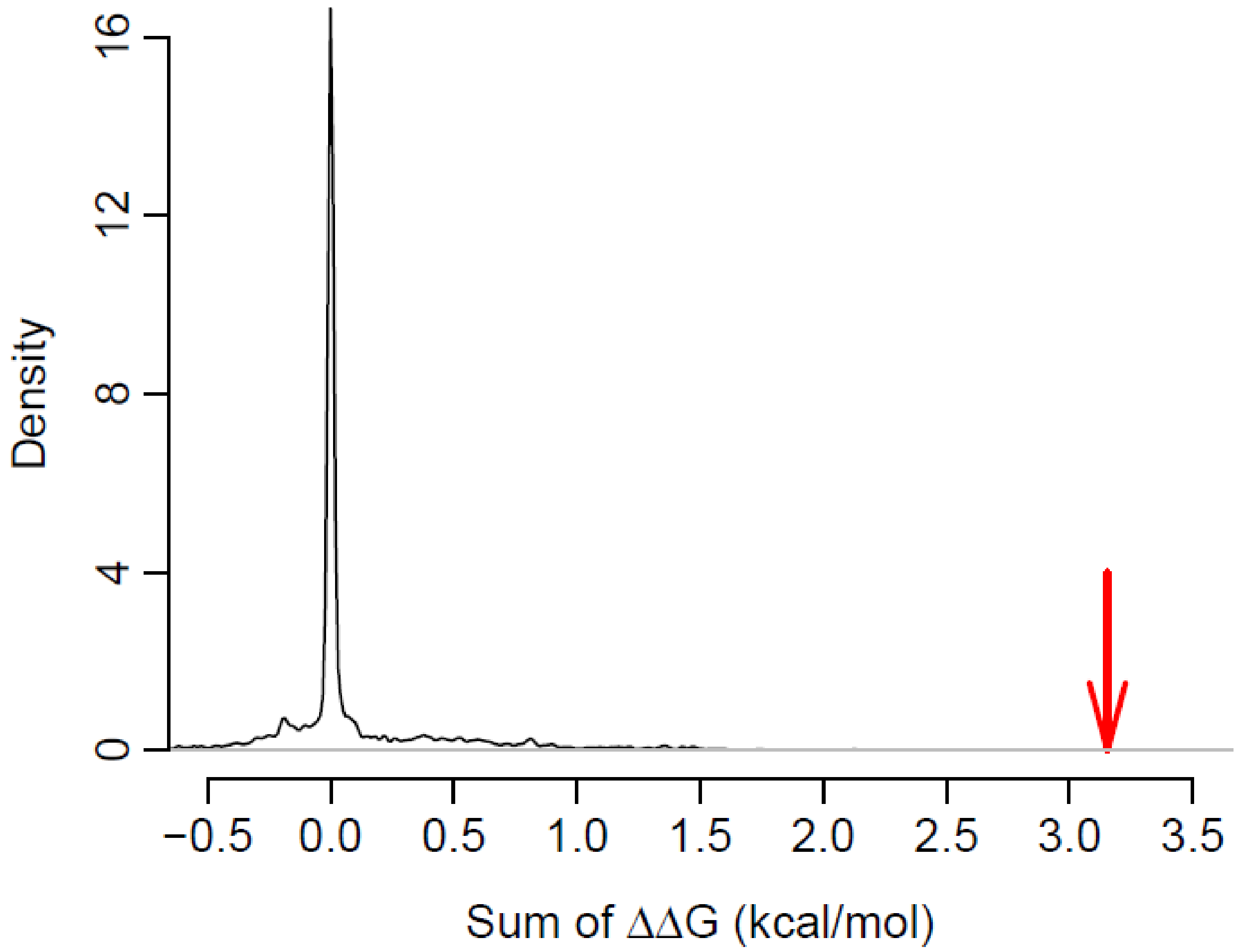

Figure S5. The distribution of the sum of local accessibility gap (ΔΔG) of 5 randomly selected windows. For each randomization, we randomly picked up 5 windows and calculated the sum of ΔΔG. Among 10,000 times of randomization, we didn't find any case with the sum of ΔΔG larger than that of the 5 clustered windows around start codon. The red arrow indicates the sum of ΔΔG of the 5 clustered windows around start codon.

Supplement Figure S6

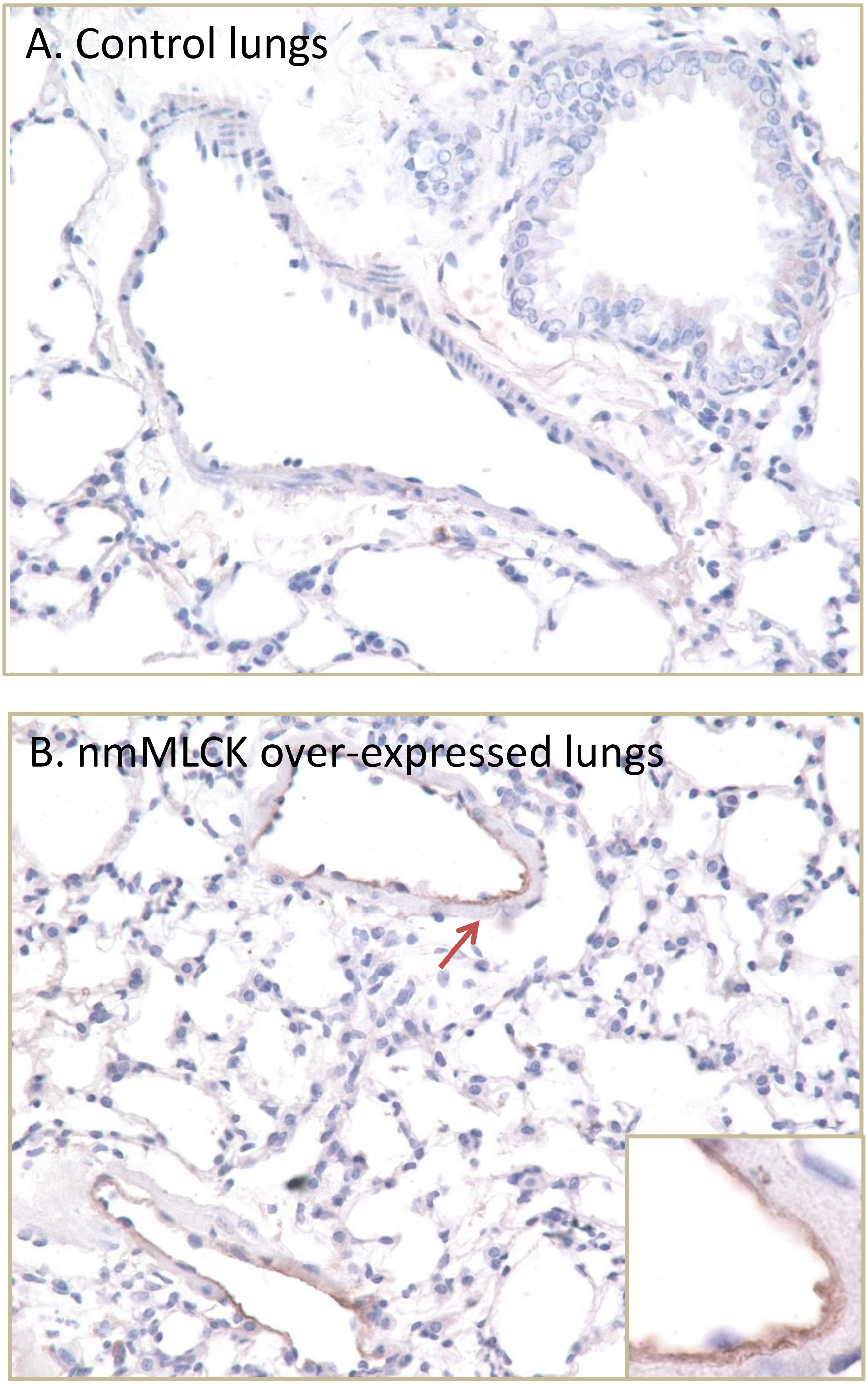

Figure S6. Immunohistochemical (IHC) analysis of nmMLCK expression in murine lung tissues (nmMLCK$^{-/-}$) after transfection of nmMLCK plasmid with liposome delivery system. The area pointed by the red arrow is amplified in the inlet.

Supplement Figure S7

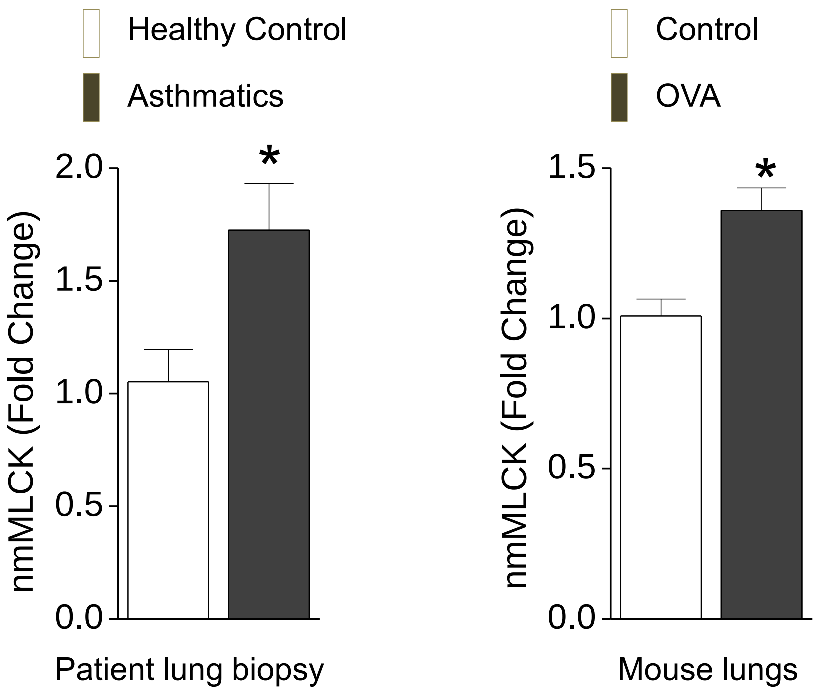

Figure S7. nmMLCK mRNA expression in human lung biopsy and mouse lung tissues. *, p<0.05 compared to control.
